# Gender disparities in multiple sclerosis research and leadership: A Colombian perspective

**DOI:** 10.3389/fneur.2022.1020051

**Published:** 2022-10-18

**Authors:** Adriana Casallas-Vanegas, Claudia Guío-Sánchez, María I. Reyes-Mantilla, Carolina Restrepo-Aristizábal, Angela Navas-Granados, Alejandra Guerrero-Gómez, María I. Zuluaga

**Affiliations:** ^1^Cayre Multiple Sclerosis Center, Bogotá, Colombia; ^2^Neurology and Neuroimmunology Department, Instituto Nacional de Neurología y Neurocirugía, Mexico City, Mexico; ^3^Multiple Sclerosis Center, Hospital Universitario Nacional de Colombia, Bogotá, Colombia; ^4^Neurology Department, Hospital Universitario Mayor Méderi, Bogotá, Colombia; ^5^Division of Neuroimmunoloy and Neuroinfectious Diseases, Johns Hopkins University, Baltimore, MD, United States; ^6^Multiple Sclerosis Center Fundación Instituto Neurológico de Colombia, Universidad CES, Medellín, Colombia; ^7^Clínica CES, Universidad CES, Medellín, Colombia; ^8^Mutilple Sclerosis Center Medicarte, Medellín, Colombia

**Keywords:** central nervous system diseases, demyelinating disorders, equity, diversity and inclusion, workforce diversity, gender gap, multiple sclerosis, women

## Abstract

**Background:**

The neurological academic field is an illustrative example of persistent gender-related disparities reflected in compensation, funding, leadership, promotion, publishing, and recognition. Several studies indicate that neurology is one of the most underrepresented specialties with female physicians as first authors, but also has one of the highest gender payment gaps. Neglecting the role of women in academic leadership positions hinders the visibility and recognition of research and leadership in multiple sclerosis (MS). Increasing diversity within academia has positive effects, such as widening focus and expanding the plurality of research outputs. The gender gap and visibility of female MS clinicians and researchers remains an unexplored research topic in our country despite the rising number of female neurologists.

**Objective:**

This study aims to establish the gender distribution between researchers and clinical neurologists in multiple sclerosis in Colombia and raise awareness about gender disparities in this area.

**Methods:**

We applied a cross-sectional survey study of Colombian neurologists and neurology residents currently members of the Colombian Neurology Association. Mean and standard deviation (SD) were used for quantitative variables and frequency for qualitative variables. To evaluate the influence of gender, logarithmic regression was used. Data were analyzed in SPSS 26.

**Results:**

A total of 201 participants agreed to complete the survey, most of whom were female (*n* = 135, 67.2%). All the Colombian regions were represented in the survey. Of those surveyed, 31.5% (*n* = 64) had an interest in demyelinating diseases and MS, of which 46.8% (*n* = 30) were female. Of the women with MS training, only 50% (*n* =5) had more than three publications as the first author of a scientific article compared to men (*n* = 5, 83%). After adjusting the number of publications by gender, there were no significant differences between men and women (median 2.0[2, 1.21] vs. 2[2, 0.5], *p* = 0.904). However, only 16.6% (*n* = 5) of women had a visible academic, leadership, or teaching position compared with men 75.7% (*n* = 25). When adjusting the salary income by gender, we found a statistically significant difference between women and men (median 2.0 [5, 1.47] vs. 3 [5, 1.65], *p* = 0.006). Women in MS earned between USD 2,500 and 3,800 per month; while men earned between USD 3,800 to 5,070.

**Conclusion:**

Despite a higher number of female neurologists trained in MS in Colombia, our data suggest considerable differences and gender gaps with regard to diverse opportunities at the academic, salary promotion, leadership, teaching, and recognition levels between male and female MS neurologists.

## Introduction

Today, there is increasing awareness of gender disparities in academic medicine, which is also seen in neurology and multiple sclerosis (MS) research (1). Nevertheless, despite the fact that there has been a substantial increase in women pursuing careers within the neurology field, multiple research projects have shown that female neurologists have high rates of burnout and gender disparities (2).

From an academic and research point of view, women are less likely to participate in collaborations that lead to publication. Several studies across the world show that women have been historically underrepresented as first and last authors in publications and from phase 3 clinical trials in MS, sponsored by pharmaceutical companies (3). Another study, among high-profile neurology journals, showed that only 25% of peer-reviewed articles had a woman as a first author (4). At the same time, recent data published by the American Academy of Neurology (AAN) revealed that neurology has one of the highest gender pay gaps across medical specialties (5) which may indeed affect the decision of women to enter the academic field in neurology (6).

Neglecting the role of women in academic leadership positions hinders the visibility and recognition of research and leadership in MS. This phenomenon could exclude or delay important contributions to science. This can affect progress toward a better understanding of the pathogenesis, treatment, and prognosis of neurological diseases including MS (7). Increasing diversity within academia has positive effects, such as widening focus and expanding the plurality of research outputs. The gender gap and visibility of female MS clinicians and researchers remains an unexplored research topic in our country in spite of the increased number of female neurologists.

This study aims to establish the gender distribution among researchers and clinical practitioners in multiple sclerosis in Colombia and raise awareness about gender disparities in this area.

## Methods

We performed a cross-sectional survey study of Colombian neurologists and neurology residents currently members of the Colombian Neurology Association who agreed to participate in our research. Questions included demographics, the field of interest in neurology, mentoring during residency, publication authorship, income, and participation in pharmaceutical-industry events. Gender was categorized in a binary manner as male and female. Mean and standard deviation (SD) were used for quantitative variables and frequency for qualitative variables. To evaluate the influence of gender, linear or logarithmic regression was used as appropriate. *P* ≤ 0.05 were considered statistically significant. Data were analyzed using IBM SPSS Statistics 26.

## Results

**Figure 1:**
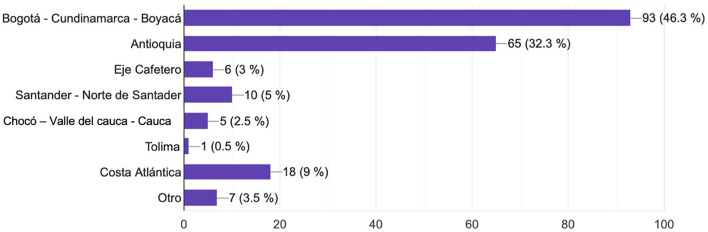
Colombian regions represented on the survey. All Colombian departments were represented in the survey.

**Figure 2:**
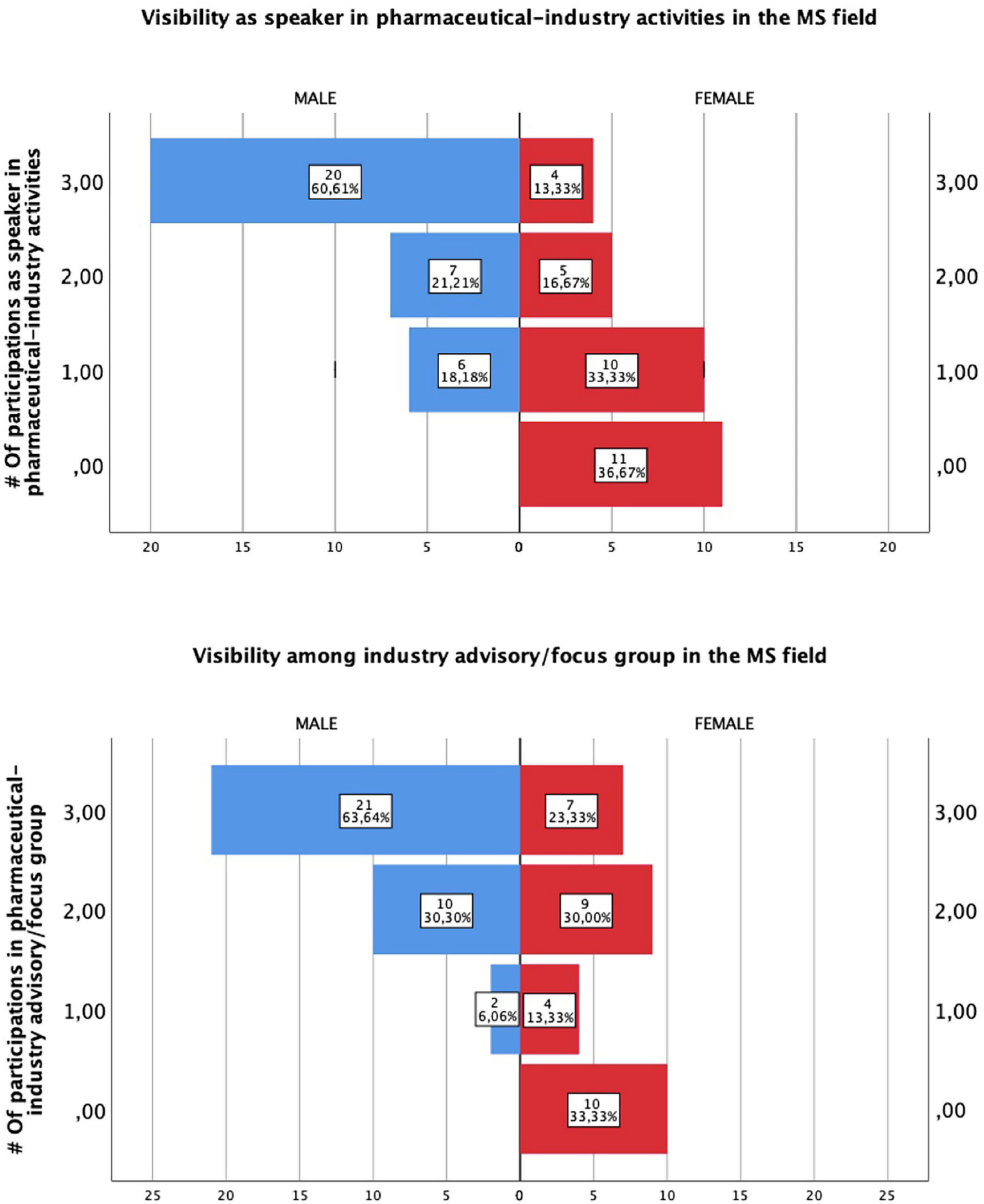
Gender visibility gaps in the MS field. Activities were calculated in the last year. MS. Multiple Sclerosis.

A total of 201 participants agreed to complete the survey, most of whom were female (*n* = 135, 67.2%) with men being 32.8% (*n* = 65) (Table 1). At least half of our participants' ages ranged from 31–40 y old (*n* = 104, 51.7%). All the Colombian regions were represented in the survey (Figure 1). Of those surveyed, 31.5% (*n* = 64) had an interest in demyelinating diseases and MS, of whom 46.8% (*n* = 30) were female. In subgroup analysis, we found that a subspecialty or master's degree in demyelinating diseases (*n* = 16) was completed by 62.5% (*n* = 10) of women and 37.5% of men (*n* = 6). Of the women with MS training, only 50% (*n* = 5) had more than three publications as the first author of a scientific article compared to men (*n* = 5, 83%). After adjusting the number of publications by gender, there were no significant differences between men and women (median 2.0[2, 1.21] vs. 2[2, 0.5], *p* = 0.904). However, only 16.6% (*n* = 5) of women had a visible academic, leadership, or teaching position compared with men 75.7% (*n* = 25). Regarding participation in industry activities as an advisory or focus group, female representation is evidently less than male (median 2.0[3, 1.19] vs. 2[2, 0.61], *p* = 0.03). When we compared female and male participation as a speaker, we found a significant difference in this field (median 2.0[3, 1.04] vs. 2[2, 0.79], *p* = 0.000) (Figure 2).

**Table 1:** Demographics.

**Gender n, (%)**	**Female 135 (67,2)**	**Male 66 (32,8)**	
**Age range (%)**			
20 to 30 years	24 (17.7)	10 (15.1)	
31 to 40 years	78 (57.5)	26 (39.3)	
41 to 50 years	21 (15.5)	20 (30.3)	
51 to 60 years	8 (5.9)	4 (6,06)	
> 60 years	4 (2,96)	6 (9.09)	
Interest in demyelinating diseases and ms n, (%)	30 (47,6)	33 (52,3)	
Subspecialty or master's degree in demyelinating diseases n, (%)	10 (62,5)	6 (37,5)	
**Publication as first author in neurology n, (%)**			***(P** **=** **0.904)***
0	4 (13.3)	3 (9.09)	
1	6 (20)	4 (12.1)	
2	5 (16.6)	11 (33,3)	
3	9 (30)	6 (18.1)	
>4	6 (20)	9 (12.1)	
Publication as first author in subspecialty or master's degree in demyelinating diseases n, (%)	5 (50)	5 (83,3)	***(P** **=** **0.18)***
**Salary income in neurology n, (%)**			
USD 1,800 to 2,300	40 (29,6)	10 (15,1)	*(P **=** **0.006)**
USD 2,300 to 3,600	41 (30,3)	16 (24,2)	
USD 3,600 to 4,600	26 (19,2)	13 (19,6)	
USD 4,600 to 5,700	14 (10,3)	10 (15,1)	
>USD 5,700	3 (2,2)	9 (13,6)	
No response	11 (8,14)	8 (12,1)	
**Salary income in ms (range)n, (%)**			
USD 1,800 to 2,300	6 (20)	0 (0)	***(P** **=** **0.21)***
USD 2,300 to 3,600	12 (40)	11 (33,3)	
USD 3,600 to 4,600	8 (26,6)	14 (42,4)	
USD 4,600 to 5,700	4 (13,3)	4 (12,1)	
>USD 5,700	0 (0)	4 (12,1)	
Mentorship in neurology educational program n, (%)	5 (2.5)	110 (54.7)	
**# Of participations in pharmaceutical industry advisory / focus group n, (%)**			
0	84 (62,2)	41 (62)	***(P** **=** **0.76)***
1	27 (20)	6 (9,09)	
2	11 (8,14)	5 (5,18)	
3	13 (9,62)	13 (19,6)	
**# Of participations in pharmaceutical industry advisory / focus group in ms field n, (%)**			
0	10 (33,3)	0 (0)	*(P **=** **0.03)**
1	4 (13,3)	2 (6,06)	
2	9(30)	10 (30,3)	
3	7 (23,3)	21 (63,6)	
**# Of participations as speaker in pharmaceutical industry activities in neurology n, (%)**			
0	81 (60)	31(46)	***(P** **=** **0,08)***
1	12 (8,8)	3 (4,5)	
2	12 (8,8)	8 (12)	
3	30 (22,2)	24 (36)	
**# Of participations as speaker in pharmaceutical industry activities in ms field n, (%)**			
0	11 (36,6)	0 (0)	*(P **=** **0,000)**
1	10 (33,3)	6 (18,8)	
2	5 (16,6)	7 (21,2)	
3	4 (13,3)	20 (60,6)	
Visible academic, leadership, or teaching position in ms field n, (%)	5 (16.6)	25 (75.7)	

^*^Statistically significant difference.

**Figure 3:**
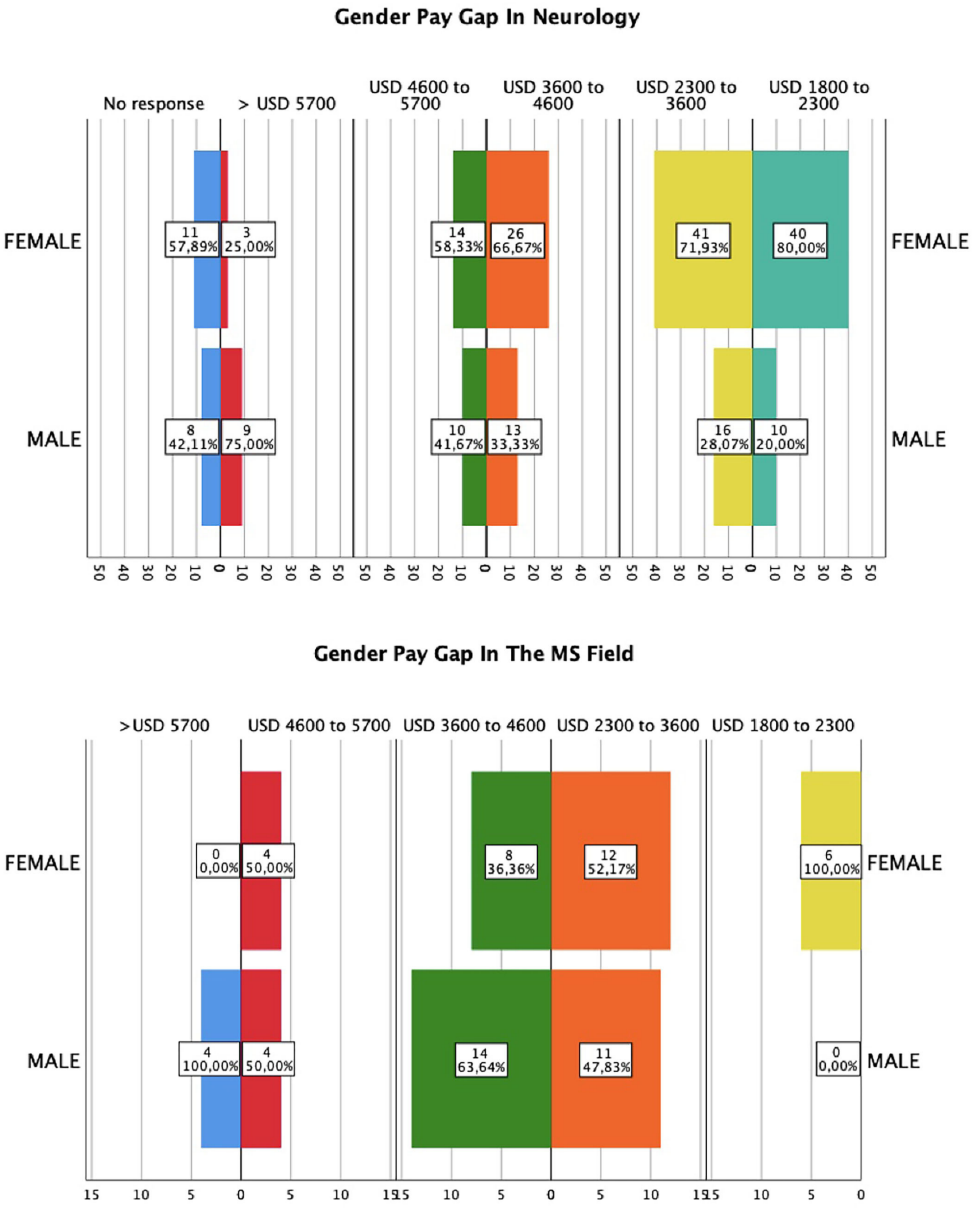
Gender pay gaps across neurology and MS field. Payment was calculated by monthly income. USD, U.S Dollar.

**Figure 4:**
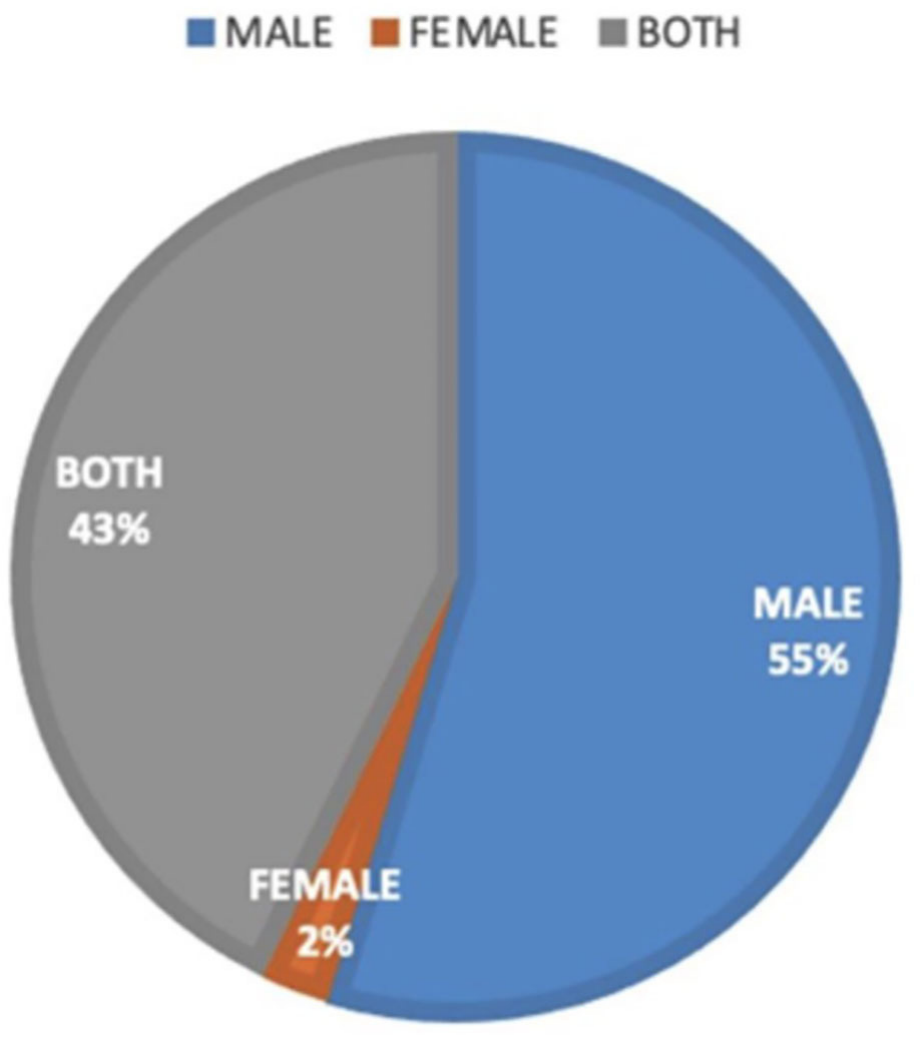
Mentorship in educational programs. Mentorship educational activities included leadership position according to gender.

When adjusting the salary income by gender, we found a statistically significant difference between women and men (median 2.0 [5, 1.47] vs. 3 [5, 1.65], *p* = 0.006). Women in MS earned between USD 2,500 and 3,800 per month, while men earned between USD 3,800 to 5,070 (Figure 3). It was identified that during the training in neurology, the mentors tended to be male (*n* =110, 54.7%), while female mentors accounted for only 2.5% of the cases. In the distribution of mentorship in educational programs in Colombia, it can be observed that only 2% of the mentorship is exclusively by women (Figure 4).

## Discussion

Our data demonstrate considerable differences in research inclusion, academic participation, and representation between women and men in the MS field in Colombia, reflecting the current landscape of the scientific community in other parts of the world. These differences exist in multiple areas, including visibility at conferences, authorship of journal articles, and participation in education and mentoring activities, with potential implications for both research priorities and the care of people with MS.

Although multiple sclerosis is a disease that affects women more frequently than men, patient care and clinical research in this field have traditionally been led by men (8). This is corroborated in the present study, which highlights the current situation of women in neurology and MS in Colombia.

In Colombia, a study published in 2012 indicated that there were 295 certified neurologists, of whom the majority were men (*n* = 221, 75%) and who were located in the main cities, Bogotá and Medellín. Recent data from 2020 (not yet published) calculates a total of 547 neurologists. Even with the higher number of men in the neurology field, the survey was predominantly completed by women, which may reflect the lack of visibility and awareness of the gender gap in our country (9).

Moreover, despite the rising proportion of women in the field with postgraduate training in MS, their academic and leadership positions continue to be scarce; salaries are still inferior compared to men's and they are underrepresented in scientific literature. These results are aligned with those published in other countries (10). Data from the Association of American Medical Colleges showed that men outnumbered women at all levels in academic neurology programs (professor: 13%; associate professor: 30%; assistant professor: 43%; and instructor: 40%). Also, neurology has the highest salary gap of all medical specialties among men and women with a difference of USD 37,000/year (11); this difference was also noted in our survey. The spectrum of inequities among female neurologists is underrepresented in leadership positions, and academic promotion, especially at the professor level and in publishing literature (6).

Research authorship is another intriguing gender gap. A study was done in Canada and the United States examined the authorship trends in neuroscience literature and found that men dominate first and last authorship. Furthermore, they found that men as the last authors were more likely to be published (10). The gender gap is also apparent within the pharmaceutical companies, which lead phase 3 trials in multiple sclerosis. A recent study stated that in 77% of the trials, the first author was male, compared to only 23% female (12); this disproportion of male vs. female speakers was also found to be true at satellite symposia in MS meetings (8) and further supported by our study.

In high-income countries with considerable access to research, the reasons for gender disparities at the academic level are poorly understood; some hypotheses are: less funding for independent research for female investigators, women are less interested in manuscript production, men negotiate for better positions in the authorship process, and there is a predilection for men in the review process (2).

Our study is the first to explore these gender disparities among Colombian MS female neurologists. In developing countries, the causes of the gender gap in science are diverse, ranging from the influence of discrimination and gender stereotypes, socialization, future expectation, and self-esteem, to the influence of gender roles and family life (13). Our article opens the discussion to explore the influence of all these dimensions that remain underexplored, particularly in Latin-American regions and in our neurology discipline.

In Latin America, there is no clear information about this gender situation; however, there is a low representation of women in MS associations, specifically in Latin America. An example is the Latin American Committee for Treatment and Research in Multiple Sclerosis (LACTRIMS), where it is observed that there is no woman as an authority, and women represent only 20% of the entire Latin American committee, which should be taken into account for future inclusions and adjustments (14).

Despite the uniqueness of this study of female neurologists in Colombia, our study has some limitations. Even though there was a representation of all the territories in the country, we received information from only 201 of the 550 neurologists registered in Colombia, which suggests that there may have been underrepresented neurologists who do not have access to social media (e.g., WhatsApp groups) or electronic platforms through which the survey was socialized. Another limitation is that variables–such as the number of hours worked by each gender and time spent at work–were not considered in this study and should be included in any future analysis.

Quantification and recognition of the gender gap is the first step in taking action. Initiatives in the field of MS such as the one led by International Women in MS (IWIMS) (12) seek to promote mentoring and training networks for women in clinical practice and research. A call to action should focus on the convening power of the pharmaceutical industry, academic leaders, editorial committees, and scientific societies so that greater representation of women and men is considered within their inclusion policies.

Nonetheless, it is important to highlight the current actionable efforts to narrow the gap. Ten years ago, there were no women as opinion leaders in the MS field in Colombia. This initiative is, in fact, a step forward to achieving a more equal and just environment for the practice of neurology in our country.

## Conclusion

Our data highlight considerable differences between male and female physicians' opportunities in MS. Despite a higher number of female physicians trained in MS in Colombia, they tend to have lower incomes and only a minority have had a visible academic, leadership, or teaching position when compared to their male counterparts. We believe that it is time for the MS community to work tirelessly to achieve equal, equitable participation of women at all levels throughout the world and develop mechanisms to identify inequalities, and thus, encourage efforts to raise awareness of gender-gap disparities.

## Data availability statement

The original contributions presented in the study are included in the article/supplementary material, further inquiries can be directed to the corresponding author/s.

## Ethics statement

The studies involving human participants were reviewed and approved by the Ethics Committee of the CAYRE MS Center. Written informed consent from the patients/participants or patients/participants' legal guardian/next of kin was not required to participate in this study in accordance with the national legislation and the institutional requirements.

## Author contributions

AC-V and MZ: writing-review and editing. CR-A and AC-V: writing-original draft and formal analysis. CG-S: editing. CG-S and MR-M: investigation. AG-G, MR-M, CG-S, and AN-G: data curation. MZ: conceive and design the survey and collected data. All authors contributed to the article and approved the submitted version.

## Conflict of interest

The authors declare that the research was conducted in the absence of any commercial or financial relationships that could be construed as a potential conflict of interest.

## Publisher's note

All claims expressed in this article are solely those of the authors and do not necessarily represent those of their affiliated organizations, or those of the publisher, the editors and the reviewers. Any product that may be evaluated in this article, or claim that may be made by its manufacturer, is not guaranteed or endorsed by the publisher.
